# The Potential Impact of Serum Sodium and Potassium Levels on Sensorineural Hearing Loss and Tinnitus

**DOI:** 10.3390/jcm15062225

**Published:** 2026-03-15

**Authors:** Stefani Maihoub, Panayiota Mavrogeni, Aphrodite Mavrogenis, András Molnár

**Affiliations:** 1 Maihoub ENT Clinic, Aliakmona Street 16, Limassol 3117, Cyprus; stephaniemaihoub@gmail.com; 2 Faculty of Health Sciences Doctoral School, University of Pécs, Vörösmarty Street 4, 7621 Pécs, Hungary; mavrogeni.panayiota@edu.pte.hu; 3 Bajcsy–Zsilinszky Hospital, Maglódi út 89-91, 1106 Budapest, Hungary; m.aphrodite94@gmail.com; 4 Opera Clinic, Protone Audio Kft., Lázár u 4, 1065 Budapest, Hungary

**Keywords:** tinnitus, sensorineural hearing loss, serum sodium levels, serum potassium levels, hearing loss severity, tinnitus intensity

## Abstract

**Objectives:** This study aimed to analyse the impact of serum sodium and potassium levels on sensorineural hearing loss and tinnitus. **Material and Methods:** A total of 616 individuals participated—248 with sensorineural hearing loss and tinnitus, 136 with tinnitus only, and 232 controls. All patients received thorough examinations by specialists in otorhinolaryngology and audiology. Additionally, all participants underwent laboratory testing. **Results:** Serum sodium levels were slightly elevated in the tinnitus group with sensorineural hearing loss compared to both the tinnitus group and the control group, although these differences were not statistically significant (*p* = 0.27 and *p* = 0.89). Additionally, this trend was not observed when comparing the tinnitus group to the control group (*p* = 0.32). The serum potassium levels did not show a significant difference among the three groups (*p* = 0.155). In the group experiencing sensorineural hearing loss and tinnitus, a significant positive correlation was found between serum sodium levels and the onset of sensorineural hearing loss (*p* = 0.000, rho = 0.223). Additionally, there was a significant correlation between age and the onset of symptoms (*p* = 0.000, rho = 0.235). No significant correlations were found regarding serum potassium levels. Patients with hearing levels exceeding 40 dB exhibited slightly higher serum sodium levels (*p* = 0.56). Both the groups with tinnitus and sensorineural hearing loss, as well as the tinnitus-only group, showed a trend indicating that higher serum sodium levels were associated with greater tinnitus intensities (*p* = 0.43 and *p* = 0.62, respectively). A logistic regression analysis indicated that the development of sensorineural hearing loss and tinnitus was significantly associated with changes in serum sodium levels (*p* = 0.030; OR: 0.186, 95% CI = 0.027–5.550). Additionally, serum sodium levels were found to be significant predictors of more severe hearing loss, defined as hearing levels exceeding 40 dB (*p* = 0.019; OR: 1.950, 95% CI = 0.504–7.540). **Conclusions:** The findings of this investigation suggest that changes in serum sodium levels may influence the development and severity of tinnitus and sensorineural hearing loss. Further studies involving more cases are needed to solidify these results.

## 1. Introduction

Sensorineural hearing loss (SNHL) and tinnitus are two of the most common and debilitating auditory disorders worldwide. They significantly impact communication, cognitive function, psychological well-being, and overall quality of life [1]. SNHL results from dysfunction in the hair cells of the inner ear, the auditory nerve, or the central auditory pathways. This condition results from a complex interplay of factors, including ageing, noise exposure, genetic predisposition, exposure to ototoxic substances, infections, autoimmune diseases, and metabolic disorders [2]. Tinnitus, characterised by the perception of a phantom sound without any external acoustic stimulus, often accompanies hearing loss. However, it can also occur in individuals with normal hearing thresholds [3]. The co-occurrence of tinnitus and SNHL indicates that there may be shared, yet not fully understood, physiological mechanisms at play. These likely involve damage to the peripheral cochlea and subsequent maladaptive neuroplastic changes in the central auditory system [4,5]. Despite extensive research, the exact mechanisms that initiate and sustain these auditory problems remain unclear. This uncertainty drives the investigation of new contributing factors, including issues related to systemic metabolism and ionic balance.

The microenvironment of the inner ear relies on a precise electrochemical gradient for effective auditory transduction. Maintaining specific ion gradients, particularly sodium (Na+) and potassium (K+), along with proper fluid composition, is crucial [6]. The endocochlear potential, a distinct voltage of +80 mV within the scala media, is primarily generated by the active transport of potassium ions (K+) through the stria vascularis [7,8]. This potassium-rich fluid, known as endolymph, is essential for depolarising hair cells in response to sound vibrations. Although sodium ions (Na+) are present in lower concentrations in the endolymph, they are vital for generating action potentials in spiral ganglion neurons and for maintaining overall fluid and osmotic balance [9]. Disruptions in serum levels of essential electrolytes can potentially disrupt cochlear homeostasis and alter neuronal excitability, which may contribute to auditory dysfunction. Recent preclinical and clinical studies have started to explore potential connections between electrolyte imbalances and auditory outcomes. For instance, animal models have shown that disturbances in potassium recycling pathways within the cochlea can lead to hearing loss and tinnitus-like symptoms [10]. Additionally, clinical reports indicate that conditions commonly associated with electrolyte disturbances, such as diabetes mellitus, have higher incidences of SNHL and tinnitus, suggesting a possible systemic link [11,12]. Hyponatraemia is linked to a variety of neurological symptoms, ranging from problems with gait to cognitive impairments, which may also affect central auditory processing [13]. On the other hand, hypernatraemia causes cellular dehydration and can increase neuronal excitability. This heightened state could theoretically lower the threshold for perceiving tinnitus or worsen hyperactivity in the auditory nerve. Additionally, both hypokalaemia and hyperkalaemia can disrupt cellular repolarisation and neurotransmitter release, potentially impacting synaptic transmission in auditory pathways [14]. Hyperkalaemia can lead to neurological symptoms such as muscle weakness, paraesthesia (tingling or numbness), and, in severe cases, even flaccid paralysis [15]. On the other hand, hypokalaemia causes progressive muscle weakness, hypokalaemic periodic paralysis, sensory disturbances, and even neuropsychiatric symptoms [16]. Potassium is essential for maintaining a normal electrolyte environment in the inner ear. Although the specific effects of potassium disturbances are not well understood, some studies have linked a high-potassium diet to a lower prevalence of hearing loss. This connection may be attributed to reduced microvascular damage to the cochlea. Additionally, a high-potassium diet is linked to increased serum aldosterone levels, which may help prevent hearing impairment by enhancing the functions of Na^+^-K^+^ ATPase and the Na^+^-K^+^-2Cl^−^ cotransporter (NKCC1) [17].

However, the current clinical literature reveals significant gaps in our understanding of how electrolytes relate to auditory function. Most studies have focused on severe electrolyte imbalances in acutely or critically ill patients, where hearing function is rarely a primary concern. Furthermore, it is still unclear how these ions might differentially influence hearing loss and tinnitus or whether they are linked to specific clinical features such as the duration of symptoms, their laterality, or their perceived impact on daily life. Addressing these questions is essential to determine whether routine serum electrolyte testing can offer prognostic or diagnostic value in audiology, as well as to explore new therapeutic approaches aimed at restoring ionic balance.

This study aimed to conduct a comprehensive analysis of the potential impact of serum sodium and potassium levels on SNHL and tinnitus. By comparing well-defined groups, we investigated the differences in serum electrolyte levels between these groups, along with the correlations between electrolyte levels and key clinical parameters, such as age of onset, hearing threshold, tinnitus intensity, and associated handicap. We hypothesised that elevated serum sodium and potassium levels would be associated with an increased likelihood of concurrent SNHL and tinnitus, more severe hearing loss, and greater tinnitus-related handicap, indicating underlying disturbances in cochlear and neural ionic homeostasis.

## 2. Materials and Methods

### 2.1. Study Groups

**Figure 1:**
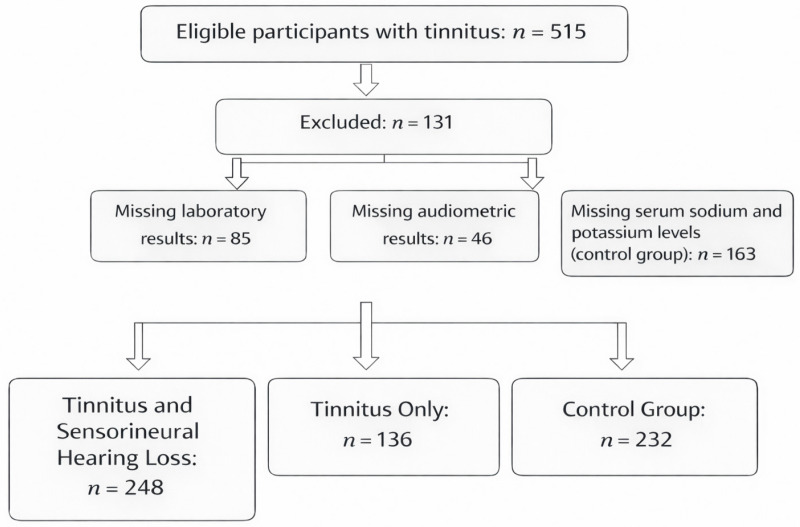
Flowchart depicting the study’s populations.

Sample size calculations were performed, determining that 223 participants were needed for the study (resulting in a total of *n* = 248 + 136 participants) and for the control group (resulting in *n* = 232 participants), with α = 0.05 and 1 − β = 0.8. A total of 616 individuals participated in this study. Among these, 248 patients experienced both SNHL and tinnitus, 136 had tinnitus without SNHL, and 232 individuals without hearing issues or tinnitus served as the control group (Figure 1). The detailed characteristics of the three groups are presented in Table 1. All patients underwent a thorough examination by otorhinolaryngologists and received tinnitus management from experienced doctors. Each patient also had a comprehensive hearing assessment as detailed below. As part of routine management, all patients underwent laboratory tests closely related to the onset of their symptoms to assess serum sodium and potassium levels. To rule out retrocochlear and intracranial lesions, all patients had a contrast-enhanced brain MRI. The inclusion criteria focused on primary tinnitus cases, which could occur with or without SNHL. Exclusion criteria included factors that could affect serum sodium and potassium levels, such as hormonal disorders (e.g., SIADH, Addison’s disease, or Cushing’s syndrome), kidney, liver, and heart problems, certain medications (e.g., diuretics, antidepressants, ACE inhibitors, laxatives, or NSAIDs), severe diarrhoea or vomiting at the time of examination, metabolic disorders (e.g., diabetes mellitus), and significant traumas or burns. All participants provided written consent to take part in the study. The investigation adhered to the Declaration of Helsinki and received approval from the Hungarian ETT TUKEB (approval number: BM/29864-1/2024, approval date: 9 December 2024).

### 2.2. Hearing Assessments

Before conducting audiometric examinations, each patient underwent micro-otoscopy, tympanometry, and acoustic reflex testing. Following these assessments, a qualified audiological assistant performed pure-tone audiometry and tinnitus matching for every patient. The audiometric evaluations took place in a soundproof booth. Pure-tone air conduction was measured across frequencies ranging from 125 to 8000 Hz using headphones, while bone conduction was assessed from 250 to 4000 Hz with a mastoid vibrator. Masked bone conduction measurements were conducted as needed. The pure-tone audiograms were then manually constructed. Tinnitus matching began with pitch matching, aiming to identify the most accurate frequency range of the tinnitus. Once this was completed, the intensity of the tinnitus was measured in 1 dB increments at the identified frequency during the intensity matching process. The results were manually recorded on audiograms. SNHL was defined according to the 1995 recommendations from the Committee on Hearing and Equilibrium of the American Academy of Otolaryngology–Head and Neck Surgery [18]. Audiometer calibration was conducted according to both local and international standards, including ISO 389-1:2017 [19], which specifies the reference zero for calibration, particularly for pure-tone air conduction, and IEC 60645-1 [19], which outlines international standards for pure-tone audiometers.

### 2.3. Self-Reported Tinnitus Severity

Self-reported tinnitus severity was measured using the Tinnitus Handicap Inventory (THI) [20]. The THI assesses the impact of tinnitus on daily functioning across three scales: functional, emotional, and catastrophic. Patients respond to each question with either ’yes’ (4 points), ’sometimes’ (2 points), or ’no’ (0 points). The total THI score, which was used in this study, is calculated by summing the points from the three scales. Based on the total THI, tinnitus handicap can be categorised into five levels: no handicap (0–16 points), mild handicap (18–36 points), moderate handicap (38–56 points), severe handicap (58–76 points), and catastrophic handicap (78–100 points). All patients completed the THI in Hungarian.

### 2.4. Laboratory Testing

After obtaining consent, patients were asked to provide blood samples after fasting overnight. Blood samples were collected in native serum tubes. These samples were then stored and sent to the laboratory, where serum sodium and potassium levels were analysed. The laboratory validated the results and sent them to the managing doctors, who carefully reviewed the findings. In this investigation, serum sodium levels were categorised as follows: low (under 135 mmol/L), normal (135–145 mmol/L), and high (above 145 mmol/L). For serum potassium levels, the categories were defined as low (under 3.5 mmol/L), normal (3.5–5.2 mmol/L), and high (over 5.2 mmol/L).

### 2.5. Statistical Analysis

All statistical analyses were performed using IBM SPSS version 25 software (IBM Corporation, Armonk, NY, USA). Three methods were applied to handle outliers. The first method was removal (trimming), which was used when the outlier was confirmed to be a measurement error or invalid data; these values were simply removed. The second method was correction, which involved adjusting the outlier value rather than deleting it if an error was identified. The third method was retention, where outliers that represented natural variation or were essential for understanding the phenomenon were kept in the analysis. Since the study population was strictly selected, as indicated in the flow chart diagram, all necessary data was available, resulting in no missing data. The Shapiro–Wilk test indicated that the data did not follow a normal distribution. As a result, median values and their interquartile ranges (IQR) were used to present continuous variables. For data comparison, the Mann–Whitney *U* test and the Kruskal–Wallis test were utilised. For analysing potential correlations, Spearman’s correlation test was used. Additionally, a multinomial logistic regression model was applied. The multinomial logistic regression model considered serum sodium levels outside the normal range as a factor for predicting the occurrence of tinnitus and SNHL, tinnitus severity, more severe tinnitus, and chronic or bilateral symptoms, with each case adjusted for age and sex. A significance level of *p* < 0.05 was consistently maintained throughout all analyses.

## 3. Results

The study population’s basic parameters are outlined in Table 1.

According to Table 1, the two study groups did not show statistically significant differences in age and sex when compared to the control group, which confirms the comparability of the two groups. However, patients experiencing tinnitus without SNHL were slightly younger than those with both tinnitus and SNHL (*p* < 0.00001 *). Participants who experienced only tinnitus had a significantly shorter onset of symptoms (*p* < 0.0001 *, *z*-score: 5.62). SNHL was primarily bilateral, with median values of 45 dB on both sides, indicating moderately severe hearing loss in most cases. Tinnitus was primarily located on the left side and bilaterally, with bilateral cases predominating in the tinnitus group and left-sided cases in the tinnitus with SNHL group. This shows a statistically significant difference (*p* = 0.01 *). Significantly higher tinnitus intensities (*p* < 0.0001 *, *z*-score: 8.51) were found in the tinnitus group with SNHL; however, tinnitus frequencies did not show significant differences. Self-reported tinnitus severity scores from the THI were not significantly different between the two groups (*p* = 0.16; *z*-score: 1.38), with both groups experiencing predominantly moderately severe handicaps based on the median values.

**Figure 2:**
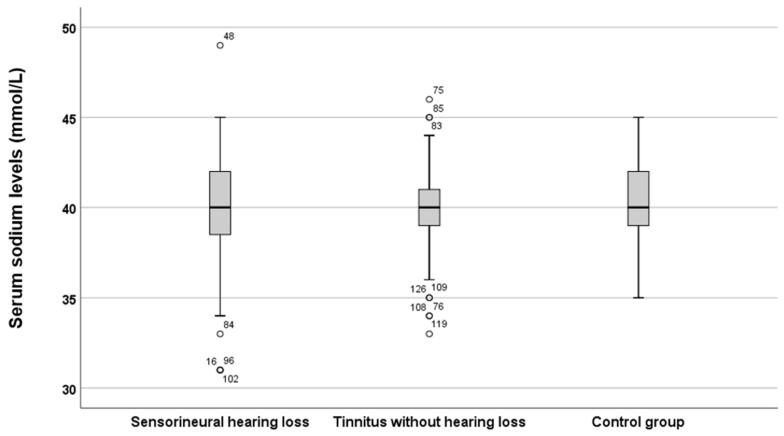
Serum sodium levels across the three groups. The boxes represent the middle 50% of the data, while the whiskers indicate the upper and lower 25%. The black line that divides each box denotes the median values. Outliers are depicted by circles and numbers. Statistical differences were analysed using the Mann–Whitney *U* test (*p* < 0.05). mmol = millimole, L = litre.

In the next step of the investigation, the sodium levels in the three groups were specifically compared. The results are depicted in Figure 2.

As shown in Figure 2, serum sodium levels were slightly higher in the tinnitus group with SNHL compared to the tinnitus and control groups. However, statistical analysis using the Mann–Whitney *U* test did not reveal significant differences (*p* = 0.27, *z*-score = 1.1 and *p* = 0.89, *z*-score = −0.13, respectively). Furthermore, when comparing serum sodium levels within the tinnitus-only group, no notable tendencies were observed, and the difference was not statistically significant (*p* = 0.32, *z*-score = 0.98).

**Figure 3:**
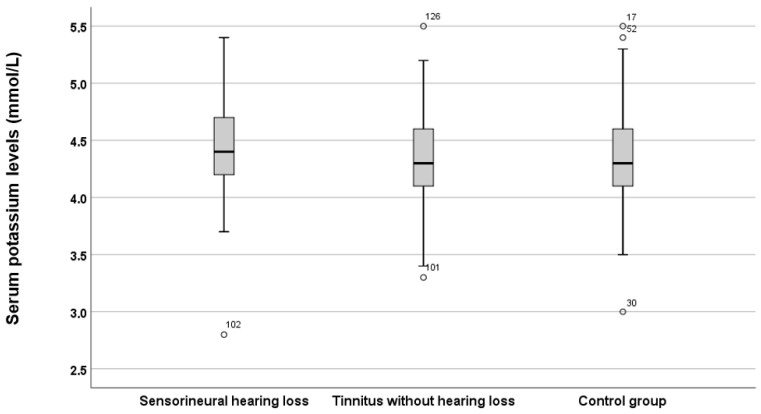
Serum potassium levels across the three groups. The boxes represent the middle 50% of the data, while the whiskers indicate the upper and lower 25%. The black line that divides each box denotes the median values. Outliers are depicted by circles and numbers. Statistical differences were analysed using the Mann–Whitney *U* test (*p* < 0.05). mmol = millimole, L = litre.

In the next step, the same analyses were conducted for serum potassium levels (Figure 3).

Figure 3 illustrates that there are no differences in serum potassium levels among the three groups, a conclusion supported by statistical analysis using the Kruskal–Wallis test (*p* = 0.155, *H* = 3.724).

**Figure 4:**
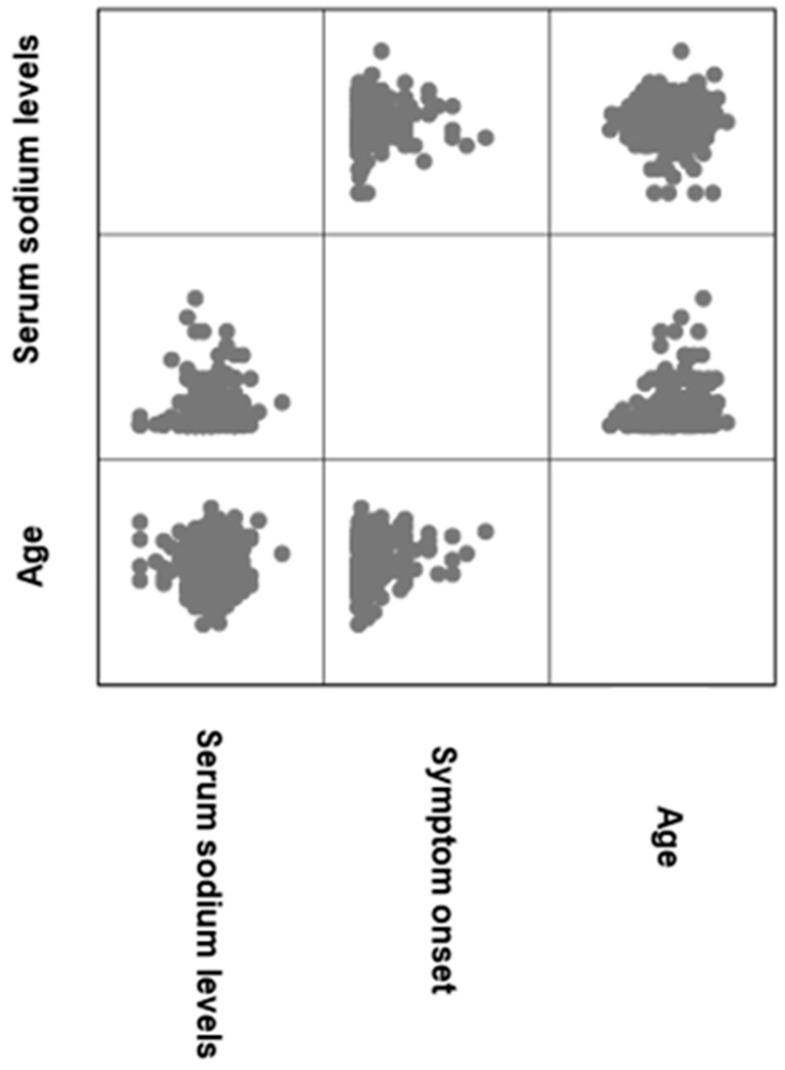
Correlation matrix of serum sodium levels and parameters in individuals with tinnitus and SNHL. SNHL = sensorineural hearing loss.

To further analyse the effects of serum sodium levels, their potential correlations with various parameters have been examined (Figure 4 and Table 2).

In the analysis of correlations within the group experiencing SNHL and tinnitus, a significant (*p* = 0.000 *) positive (rho = 0.223) correlation was found between serum sodium levels and the onset of SNHL. This indicates that higher serum sodium levels are associated with more prolonged symptoms. Additionally, there were significant (*p* = 0.000 *) positive (rho = 0.235) correlations between age and the hearing levels. Furthermore, there was a statistically significant relationship (*p* = 0.000 *) with a positive correlation (rho = 0.301) between age, onset of SNHL, and tinnitus. However, other parameters, such as tinnitus severity and intensity, did not show any significant correlations. Furthermore, when examining the correlations of serum potassium levels, no significant relationships were found; thus, these results were not included in the analysis.

**Figure 5:**
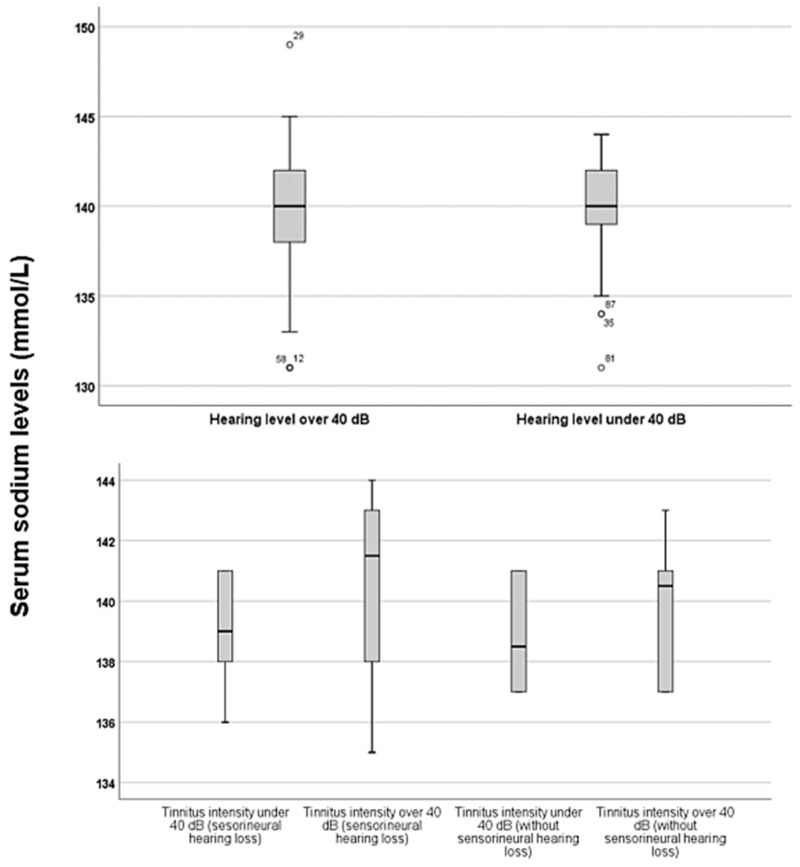
Serum sodium levels depending on hearing loss severity and tinnitus intensity. The boxes represent the middle 50% of the data, while the whiskers indicate the upper and lower 25%. The black line that divides each box denotes the median values. Outliers are depicted by circles and numbers. Statistical differences were analysed using the Mann–Whitney *U* test (*p* < 0.05). dB = decibel, mmol = millimole, L = litre.

To analyse the impact of serum sodium levels on hearing levels and tinnitus, the specific serum sodium levels based on the severity of hearing loss and the intensity of tinnitus were compared. Two groups were created for each case: one group with hearing levels above 40 dB and another with hearing levels at or below 40 dB. Additionally, tinnitus intensities were categorised into two groups: those below 40 dB and those at or above 40 dB. The results of this analysis are illustrated in Figure 5.

Figure 5 shows that patients with hearing levels exceeding 40 dB had slightly higher serum sodium levels. However, this difference was not statistically significant (*p* = 0.56, *z*-score: −0.573). In both the tinnitus and SNHL groups, as well as in the tinnitus-only group, there was only a trend suggesting that higher serum sodium levels were associated with increased tinnitus intensities. However, this difference was not statistically significant for either group (*p* = 0.43, *z*-score: −0.0781 and *p* = 0.62, *z*-score: −0.48, respectively).

According to the results presented in Table 3, changes in serum sodium levels were significant risk factors for the development of SNHL and tinnitus (*p* = 0.030 *; OR: 0.186, 95% CI = 0.027–5.550). Additionally, serum sodium levels were also significant risk factors of more severe hearing loss, defined as hearing levels over 40 dB (*p* = 0.019 *; OR: 1.950, 95% CI = 0.504–7.540). However, serum sodium levels did not significantly predict other parameters, such as the severity of tinnitus handicap, the development of chronic symptoms, or the occurrence of bilateral symptoms.

## 4. Discussion

The findings of this study present a new perspective by exploring how systemic electrolyte balance, specifically serum sodium and potassium levels, may affect the pathophysiology of SNHL and tinnitus. Although auditory transduction relies heavily on precisely regulated ion gradients within the cochlea [21], our results indicate that the overall balance of electrolytes in the body could influence, or even worsen, local cochlear vulnerabilities. This may contribute to auditory dysfunction.

In the group with SNHL and tinnitus, only a trend toward higher serum sodium levels was noted. Although this finding was not statistically significant, it supports emerging insights into systemic metabolic stress. Sodium is essential for maintaining osmotic balance, neuronal excitability, and the homeostasis of cochlear fluids [22]. Even within the normative range, a tendency toward higher sodium levels could suggest a state of low-grade hyperosmotic stress. Preclinical models have linked this condition to endothelial dysfunction, reduced cochlear perfusion, and oxidative damage to the stria vascularis and hair cells [9,23]. This is especially important as chronic subclinical stress is increasingly recognised as a factor contributing to age-related and noise-induced cochlear synaptopathy [24,25]. Consequently, our findings suggest that serum sodium may serve as a peripheral biomarker of systemic metabolic strain, potentially accelerating peripheral auditory pathology and leading to SNHL and associated tinnitus.

The notable positive correlation between serum sodium levels and the onset of symptoms in SNHL is an important observation. This indicates that the role of sodium may be progressive and related to chronic conditions rather than acute onset. This perspective aligns with the broader literature on neurodegeneration, where ongoing disturbances in sodium balance are linked to neuronal excitotoxicity and apoptotic pathways [26]. Prolonged exposure to slightly elevated sodium levels within the cochlea may impair the function of Na+/K+-ATPase, disrupt potassium recycling, or negatively affect the survival of spiral ganglion neurons [27]. The lack of a significant correlation between sodium and tinnitus-specific measures, such as the THI score, suggests that sodium’s influence is likely more peripheral, affecting the cochlear substrates that commonly, though not always, drive central tinnitus generation.

Recent logistic regression analysis reveals a significant association between serum sodium levels and the severity of hearing loss. This finding highlights the potential role of sodium (Na+) in the progression of hearing disorders. Supporting this, recent research underscores the cochlea’s vulnerability to metabolic disturbances. A study by Wu et al. suggests that even mild systemic metabolic imbalances can worsen age-related cochlear synaptopathy and hair cell loss, which are key processes in SNHL [28]. Our findings are consistent with the idea that systemic factors may influence individual susceptibility to auditory damage. Unlike the group with SNHL, the tinnitus-only group did not show any significant relationships with serum sodium levels. This observation supports the idea that sodium primarily affects cochlear integrity. In cases where there is no measurable hearing loss (as indicated by a normal audiogram), other factors—such as hidden hearing loss or central auditory gain—may play a more significant role in the perception of tinnitus [3].

The processes behind the development of SNHL are complex and not yet fully understood. Research has shown a significant link between SNHL and metabolic syndrome. For example, one study found significant associations between hearing loss and factors such as waist circumference, fasting blood sugar levels, serum HDL, serum triglyceride levels, and both systolic and diastolic blood pressure [29]. Another investigation also emphasised the significance of metabolic syndrome in relation to hearing impairment [30]. The relationship between metabolic syndrome and SNHL can be explained by various mechanisms, including oxidative stress, inflammation, microcirculation disturbances, and mitochondrial dysfunction [31]. The microvascular effects in SNHL appear to be crucial. Several mechanisms have been proposed behind them. One essential mechanism is ischemia, which refers to either an acute or chronic reduction in blood flow to the inner ear. This reduction can lead to metabolic dysfunction and damage to cochlear structures, including hair cells, the spiral ganglion, and the stria vascularis [32]. Additionally, inflammation and dysfunction of the endothelial cells in the cochlea can lead to a pro-thrombotic state, resulting in damage [33]. Reduced integrity of the blood-labyrinth barrier (BLB) can contribute to inner ear dysfunctions. The BLB is a vascular barrier primarily located in the stria vascularis of the cochlea, serving as a boundary between the blood supply and inner ear fluids. This barrier plays a crucial role in maintaining the normal ionic balance of the endolymph, specifically by ensuring high sodium levels. Consequently, this mechanism is vital for sustaining the endocochlear potential. Additionally, the BLB offers protective effects for the inner ear. Various factors, such as systemic inflammation, can disrupt the function of the BLB, leading to ionic imbalances in the endolymph [34].

In contrast, the absence of statistically significant differences in serum potassium levels is noteworthy, especially considering the well-known central role of potassium (K+) in cochlear transduction and the generation of the endocochlear potential [35]. One might expect to see a stronger correlation in this context. This finding may suggest that local potassium homeostasis in the cochlea is closely regulated and resilient to minor fluctuations in systemic potassium levels. Therefore, systemic potassium (K+) may only become a significant factor in auditory pathology during severe clinical imbalances, which were excluded from the studied groups. The cochlea operates as a largely closed-loop system for K+. This is supported by clinical observations that hearing loss is not a prominent feature of chronic, stable hypo- or hyperkalaemia. Instead, auditory issues related to potassium are likely caused by direct disruptions to the local cellular mechanisms within this pathway. These disruptions may arise from mutations in ion channels, such as KCNQ4, KCNQ1, and KCNE1, or in transporters expressed in the inner ear. These mutations are associated with both syndromic and non-syndromic hereditary hearing loss [36,37].

According to our findings, sodium levels are correlated with the presence and severity of SNHL, which is a common trigger for tinnitus. However, sodium levels do not directly predict the perceived impact of tinnitus on individuals. Systemic sodium influences the peripheral cochlear pathology that often initiates tinnitus. Nevertheless, the subsequent central auditory processing, psychological reactions, and perceived severity of tinnitus are influenced by distinct neuroplastic and psychological factors, as indicated by recent studies [38,39]. Recent studies have shown that tinnitus distress is more closely associated with functional connectivity in the limbic and prefrontal networks than with peripheral auditory function, which may explain this separation [40,41].

Several limitations of this study should be acknowledged. Firstly, the study design prevents us from determining causality. We cannot ascertain whether elevated serum sodium levels contribute to SNHL or tinnitus, or if they are merely a result of shared underlying conditions. Secondly, although we established exclusion criteria, dietary and lifestyle factors, such as daily sodium and water intake or unmeasured comorbidities, could have affected electrolyte levels. Additionally, we measured total serum levels, which may not accurately reflect ion concentrations at the critical interfaces of the stria vascularis or perilymph. Future studies should track serum electrolytes alongside auditory function over time. Moreover, investigations that incorporate more direct measures of cochlear function, such as otoacoustic emissions and electrocochleography, and that explore other ions, like calcium, would provide a more comprehensive understanding of this issue. A further limitation of this study is that the tinnitus-only group was slightly younger. This is consistent with clinical observations, as patients with tinnitus but no SNHL tend to be from younger age groups.

Despite these limitations, our study may have important clinical implications. The relationship between serum sodium levels and both the presence and severity of SNHL suggests that routine electrolyte screening could offer valuable prognostic information for patients with auditory complaints, especially those experiencing progressive or unexplained hearing loss. Additionally, this finding encourages further investigation into whether modifying sodium levels through diet or medication could help protect or stabilise hearing.

## 5. Conclusions

The findings of this study provide clinical evidence suggesting that systemic serum sodium levels may be linked to both the presence and severity of SNHL, as well as tinnitus. In contrast, serum potassium levels did not demonstrate any such association in this study. Furthermore, the results related to sodium indicate a possible connection between systemic metabolic balance and cochlear health. These findings highlight the need for further research to determine whether serum sodium could serve as a biomarker for auditory dysfunction, which may lead to new prevention and management strategies in audiology.

## Figures and Tables

**Table 1:** Participants’ basic parameters. * Mann–Whitney *U* test, ** Chi-squared test. The significance level was established at *p* < 0.05. dB = decibel, Hz = Hertz, IQR = interquartile range, Q1 = first quartile, Q3 = third quartile, SNHL = sensorineural hearing loss, THI = Tinnitus Handicap Inventory. In the table, ‘a’ represents the group with SNHL and tinnitus, ‘b’ represents those with tinnitus without SNHL, and ‘c’ denotes the control group. “a-b” refers to the *p*-value comparing the groups of SNHL and Tinnitus, as well as the Tinnitus group without SNHL. “a-c” refers to the *p*-value comparing the groups of SNHL and Tinnitus, as well as the control group. “b-c” refers to the *p*-value comparing the Tinnitus group without SNHL, as well as the control group.

Parameter	^a^ SNHL and Tinnitus (*n* = 248)	^b^ Tinnitus Without SNHL (*n* = 136)	^c^ Control Group (*n* = 232)	*p*-Values
Age (median years; IQR, Q1–Q3)	57; 21 (47–68)	43; 17.5 (32.5–50)	54; 10 (51–61)	^a-b^ *p* < 0.00001 * ^a-c^ *p* = 0.24 * ^b-c^ *p* = 0.73 *
Sex (men/women)	97/151	58/78	110/122	^a-b^ *p* = 0.45 ** ^a-c^ *p* = 0.081 ** ^b-c^ *p* = 0.43 **
Symptom onset (median months; IQR, Q1–Q3)	24 (44; 4–48)	7 (11.5; 2–13.5)		*p* < 0.0001 *
SNHL				
right, *n* (%)	47 (19%)			
left, *n* (%)	46 (18.5%)			
bilateral, *n* (%)	155 (62.8%)			
Hearing level (median dB; IQR, Q1–Q3)				
right	45 (25; 30–55)			
left	45 (20; 35–55)			
Tinnitus location				
right, *n* (%)	63 (25.4%)	40 (29.4%)		*p* = 0.01 **
left, *n* (%)	87 (35%)	29 (21.3%)		
bilateral, *n* (%)	98 (39.6%)	67 (49.3%)		
Tinnitus intensity				
right (median dB; IQR, Q1–Q3)	40; 30 (6–30)	20; 15 (10–25)		*p* < 0.0001 *
left (median dB; IQR, Q1–Q3)	40; 20 (30–50)	17.5; 15 (10–25)		*p* < 0.0001 *
Tinnitus frequency				
right (median Hz; IQR, Q1–Q3)	4000; 6500 (1000–7500)	6000; 6000 (2000–8000)		*p* = 0.71 *
left (median Hz; IQR, Q1–Q3)	4000; 7000 (1000–8000)	6000; 6500 (1500–8000)		*p* = 0.4 *
Total THI scores (median; IQR, Q1–Q3)	44; 40 (26–66)	40; 34; (24–58)		*p* = 0.16 *

**Table 2:** Spearman’s correlation parameters for the group with sensorineural hearing loss and tinnitus. *p* < 0.05 *. SNHL = sensorineural hearing loss.

	rho	*p*-Value
Serum sodium levels—SNHL onset	0.223	0.000 *
Age—hearing levels	0.235	0.000 *
Age—SNHL and tinnitus onset	0.301	0.000 *

**Table 3:** Multinomial logistic regression model assessing serum sodium levels on SNHL and tinnitus parameters. Results are significant at *p* < 0.05 *. dB = decibel, CI = confidence interval, OR = Odds ratio, SNHL = sensorineural hearing loss, std. = standard, THI = Tinnitus Handicap Inventory.

Dependent	Predictor	*β*	Std. Error	*p*-Value	OR	95% CI (Lower Bound)	95% CI (Upper Bound)
Tinnitus and SNHL occurrence	Serum sodium levels	−1.680	0.774	0.030 *	0.186	0.027	5.550
SNHL severity (over 40 dB)		0.668	0.690	0.019 *	1.950	0.504	7.540
Moderately severe-severe tinnitus (total THI over 38 points)		1.679	1.093	0.125	5.360	0.629	45.670
Chronic symptoms (lasting over 3 months)		−0.337	0.696	0.628	0.714	0.183	2.793
Bilateral symptoms		0.306	0.620	0.622	1.358	0.403	4.579

## Data Availability

The data presented in this study are available on request from the corresponding author due to reasonable request.

## Abbreviations

The following abbreviations are used in this manuscript:

ACE	Angiotensin-converting enzyme
CI	Confidence interval
dB	decibel
Hz	Hertz
IBM	International Business Machines Corporation
IQR	Interquartile range
KCNE1	Potassium Voltage-Gated Channel Subfamily E Member 1
KCNQ1	Potassium Voltage-Gated Channel Subfamily Q Member 1
KCNQ4	Potassium Voltage-Gated Channel Subfamily Q Member 4
L	Litre
mmol	Millimole
MRI	Magnetic resonance imaging
NKCC1	Na-K-2Cl cotransporter-1
NSAID	Non-steroidal anti-inflammatory drug
OR	Odds ratio
SNHL	Sensorineural hearing loss
SPSS	Statistical Package for the Social Sciences
THI	Tinnitus Handicap Inventory

## References

[1] Garavello W., Schlee W., Langguth B., Gallus S. (2022). Global Prevalence and Incidence of Tinnitus: A Systematic Review and Meta-analysis. JAMA Neurol..

[2] Förster C.Y., Shityakov S., Stavrakis S., Scheper V., Lenarz T. (2025). Interplay between noise-induced sensorineural hearing loss and hypertension: Pathophysiological mechanisms and therapeutic prospects. Front. Cell. Neurosci..

[3] Schaette R., McAlpine D. (2011). Tinnitus with a normal audiogram: Physiological evidence for hidden hearing loss and computational model. J. Neurosci..

[4] Henry J.A., Roberts L.E., Caspary D.M., Theodoroff S.M., Salvi R.J. (2014). Underlying mechanisms of tinnitus: Review and clinical implications. J. Am. Acad. Audiol..

[5] Liu J., Stohl J., Overath T. (2024). Hidden hearing loss: Fifteen years at a glance. Hear. Res..

[6] Wangemann P. (2002). K^+^ cycling and the endocochlear potential. Hear. Res..

[7] Vlajkovic S.M., Suzuki-Kerr H., Nayagam B.A. (2025). Cochlear Homeostasis in Sensorineural Hearing Loss: Mechanisms, Implications, and Therapeutic Prospects. Int. J. Mol. Sci..

[8] Zdebik A.A., Wangemann P., Jentsch T.J. (2009). Potassium ion movement in the inner ear: Insights from genetic disease and mouse models. Physiology.

[9] Kim S.H., Marcus D.C. (2011). Regulation of sodium transport in the inner ear. Hear. Res..

[10] Scheidt R.E., Kale S., Heinz M.G. (2010). Noise-induced hearing loss alters the temporal dynamics of auditory-nerve responses. Hear. Res..

[11] Mittal R., McKenna K., Keith G., Lemos J.R.N., Mittal J., Hirani K. (2024). A systematic review of the association of Type I diabetes with sensorineural hearing loss. PLoS ONE.

[12] Luo S., Wen J., Bao Q., Ou H., Yi S., Peng P. (2025). Association between diabetes mellitus and tinnitus: A meta-analysis. Biomol. Biomed..

[13] Giuliani C., Peri A. (2014). Effects of Hyponatremia on the Brain. J. Clin. Med..

[14] Wu J., Kaczmarek L.K. (2021). Modulation of Neuronal Potassium Channels During Auditory Processing. Front. Neurosci..

[15] Campese V.M., Adenuga G. (2016). Electrophysiological and clinical consequences of hyperkalemia. Kidney Int. Suppl..

[16] Yang Q., Guo X., Liu D. (2018). Hypokalemia Caused by Quetiapine and Risperidone Treatment in Schizophrenia: A Case Report. Shanghai Arch. Psychiatry.

[17] Jung D.J., Lee J.Y., Cho K.H., Lee K.Y., Do J.Y., Kang S.H. (2019). Association between a High-Potassium Diet and Hearing Thresholds in the Korean Adult Population. Sci. Rep..

[18] Committee on Hearing and Equilibrium (1995). Committee on Hearing and Equilibrium guidelines for the evaluation of results of treatment of conductive hearing loss. Otolaryngol.–Head Neck Surg..

[19] (2017). Acoustics—Reference Zero for the Calibration of Audiometric Equipment.

[20] Newman C.W., Jacobson G.P., Spitzer J.B. (1996). Development of the Tinnitus Handicap Inventory. Arch. Otolaryngol. Head Neck Surg..

[21] Fettiplace R. (2017). Hair Cell Transduction, Tuning, and Synaptic Transmission in the Mammalian Cochlea. Compr. Physiol..

[22] Bernal A., Zafra M.A., Simón M.J., Mahía J. (2023). Sodium Homeostasis, a Balance Necessary for Life. Nutrients.

[23] DuPont J.J., Greaney J.L., Wenner M.M., Lennon-Edwards S.L., Sanders P.W., Farquhar W.B., Edwards D.G. (2013). High dietary sodium intake impairs endothelium-dependent dilation in healthy salt-resistant humans. J. Hypertens..

[24] Kujawa S.G., Liberman M.C. (2015). Synaptopathy in the noise-exposed and aging cochlea: Primary neural degeneration in acquired sensorineural hearing loss. Hear. Res..

[25] Johannesen P.T., Buzo B.C., Lopez-Poveda E.A. (2019). Evidence for age-related cochlear synaptopathy in humans unconnected to speech-in-noise intelligibility deficits. Hear. Res..

[26] Stys P.K. (2005). General mechanisms of axonal damage and its prevention. J. Neurol. Sci..

[27] Wangemann P. (2006). Supporting sensory transduction: Cochlear fluid homeostasis and the endocochlear potential. J. Physiol..

[28] Wu P.Z., Liberman L.D., Bennett K., de Gruttola V., O’Malley J.T., Liberman M.C. (2019). Primary Neural Degeneration in the Human Cochlea: Evidence for Hidden Hearing Loss in the Aging Ear. Neuroscience.

[29] Sahni D., Bhagat S., Bhatia L., Singh P., Chawla S., Kaur A. (2024). Association Between Metabolic Syndrome and Hearing Impairment: A Study on 200 Subjects. Ind. J. Otolaryngol. Head Neck Surg..

[30] Zand V., Dadgarnia M., Baradaranfar M., Meybodian M., Vaziribozorg S., Fazilati M. (2023). The association between metabolic syndrome and the prognosis of idiopathic sudden sensorineural hearing loss. Eur. Arch. Otorhinolaryngol..

[31] Jung S.Y., Shim H.S., Hah Y.M., Kim S.H., Yeo S.G. (2018). Association of Metabolic Syndrome with Sudden Sensorineural Hearing Loss. JAMA Otolaryngol. Head Neck Surg..

[32] Ogawa H., Okada M., Shudou M., Gyo K., Hato N. (2018). Prevention of ischemia-induced hearing loss by intravenous administration of hydrogen-rich saline in gerbil. Neurosci. Lett..

[33] Cavallaro G., Pantaleo A., Pontillo V., Barbara F., Murri A., Quaranta N. (2023). Endothelial Dysfunction and Metabolic Disorders in Patients with Sudden Sensorineural Hearing Loss. Medicina.

[34] Sekulic M., Puche R., Bodmer D., Petkovic V. (2023). Human blood-labyrinth barrier model to study the effects of cytokines and inflammation. Front. Mol. Neurosci..

[35] Cazals Y., Bévengut M., Zanella S., Brocard F., Barhanin J., Gestreau C. (2015). KCNK5 channels mostly expressed in cochlear outer sulcus cells are indispensable for hearing. Nat. Commun..

[36] Kubisch C., Schroeder B.C., Friedrich T., Lütjohann B., El-Amraoui A., Marlin S., Petit C., Jentsch T.J. (1999). KCNQ4, a novel potassium channel expressed in sensory outer hair cells, is mutated in dominant deafness. Cell.

[37] Rim J.H., Choi J.Y., Jung J., Gee H.Y. (2021). Activation of KCNQ4 as a Therapeutic Strategy to Treat Hearing Loss. Int. J. Mol. Sci..

[38] De Ridder D., Vanneste S., Weisz N., Londero A., Schlee W., Elgoyhen A.B., Langguth B. (2014). An integrative model of auditory phantom perception: Tinnitus as a unified percept of interacting separable subnetworks. Neurosci. Biobehav. Rev..

[39] Sedley W. (2019). Tinnitus: Does Gain Explain?. Neuroscience.

[40] Singh A., Smith P.F., Zheng Y. (2023). Targeting the Limbic System: Insights into Its Involvement in Tinnitus. Int. J. Mol. Sci..

[41] Zhou Q., Jiang W., Sheng H., Zhang Q., Jin D., Li H., Huang M., Yang L., Ren Y., Huang Z. (2025). Does tinnitus and emotional distress influence central auditory processing? A comparison of acute and chronic tinnitus in normal-hearing individuals. PLoS ONE.

